# Global DNA methylation patterns in Barrett’s esophagus, dysplastic Barrett’s, and esophageal adenocarcinoma are associated with BMI, gender, and tobacco use

**DOI:** 10.1186/s13148-016-0273-7

**Published:** 2016-10-27

**Authors:** Andrew M. Kaz, Chao-Jen Wong, Vinay Varadan, Joseph E. Willis, Amitabh Chak, William M. Grady

**Affiliations:** 1 Gastroenterology Section, VA Puget Sound Health Care System, Seattle, WA 98108 USA; 2 Clinical Research Division, Fred Hutchinson Cancer Research Center, Seattle, WA 98109 USA; 3 Department of Medicine, University of Washington School of Medicine, Seattle, WA 98195 USA; 4 Case Comprehensive Cancer Center, Case Western Reserve University, Cleveland, OH 44106 USA; 5 Department of Pathology, Case Western Reserve University School of Medicine, Cleveland, OH 44106 USA; 6 Division of Gastroenterology, Case Western Reserve University School of Medicine, Cleveland, OH 44106 USA

**Keywords:** Barrett’s esophagus, Esophageal adenocarcinoma, DNA methylation, Obesity, Waist-to-hip ratio, Tobacco use

## Abstract

**Background:**

The risk of developing Barrett’s esophagus (BE) and/or esophageal adenocarcinoma (EAC) is associated with specific demographic and behavioral factors, including gender, obesity/elevated body mass index (BMI), and tobacco use. Alterations in DNA methylation, an epigenetic modification that can affect gene expression and that can be influenced by environmental factors, is frequently present in both BE and EAC and is believed to play a role in the formation of BE and its progression to EAC. It is currently unknown whether obesity or tobacco smoking influences the risk of developing BE/EAC via the induction of alterations in DNA methylation. To investigate this possibility, we assessed the genome-wide methylation status of 81 esophageal tissues, including BE, dysplastic BE, and EAC epithelia using HumanMethylation450 BeadChips (Illumina).

**Results:**

We found numerous differentially methylated loci in the esophagus tissues when comparing males to females, obese to lean individuals, and smokers to nonsmokers. Differences in DNA methylation between these groups were seen in a variety of functional genomic regions and both within and outside of CpG islands. Several cancer-related pathways were found to have differentially methylated genes between these comparison groups.

**Conclusions:**

Our findings suggest obesity and tobacco smoking may influence DNA methylation in the esophagus and raise the possibility that these risk factors affect the development of BE, dysplastic BE, and EAC through influencing the epigenetic status of specific loci that have a biologically plausible role in cancer formation.

**Electronic supplementary material:**

The online version of this article (doi:10.1186/s13148-016-0273-7) contains supplementary material, which is available to authorized users.

## Background

The incidence of esophageal adenocarcinoma (EAC) has been increasing in the USA for several decades for reasons that are not entirely clear but may be related to the increasing prevalence of risk factors such as obesity [1]. The precursor lesion for EAC is Barrett’s esophagus (BE), a metaplastic condition where the squamous-lined esophageal mucosa is replaced by specialized intestinal mucosa. A minority of individuals with BE will develop EAC through a progression sequence in which BE transitions to BE with low-grade dysplasia (LGD), BE with high-grade dysplasia (HGD), and ultimately to EAC [2].

It is recognized that both genetic and epigenetic alterations arise in the esophagus during the development and progression of BE and EAC [3–5]. Epigenetic alterations, primarily in the form of hypermethylated or hypomethylated CpG dinucleotides in the DNA, have been described in BE and EAC using both candidate gene approaches and microarray-based strategies. Hypermethylation of CpGs in CpG islands in promoter regions has been associated with the repression of transcription of some genes, and hypermethylation of CpGs in gene bodies is associated with increased gene expression [6, 7]. The effects of DNA methylation on the regulation of gene expression have supported the plausibility that alterations in DNA methylation can affect disease processes in people.

Aberrant DNA methylation has been shown to occur early in the BE → dysplastic BE → EAC progression sequence [8]. The aberrant methylation of numerous cancer-related genes, such as *CDKN2A*, as well as global alterations in DNA methylation has been observed in BE, and many of these epigenetic alterations are also found in dysplastic BE and EAC [8–13]. However, despite the near universal observation of altered DNA methylation in BE and EAC, the mechanisms driving aberrant DNA methylation in the esophagus, as in most other pre-neoplastic and neoplastic tissues, remain elusive.

The risk of developing BE and/or EAC is associated with specific demographic and behavioral factors, including obesity/elevated body mass index (BMI) and tobacco use [14, 15]. Numerous mechanisms through which these factors may affect BE and/or EAC formation have been proposed [16, 17]; however, no assessment of effects on the epigenome in the esophagus has been made to date. There is evidence that certain environmental, behavioral, and demographic factors can influence the epigenetic state, which suggests that the behavioral factors associated with BE and EAC may act by inducing alterations in the methylation status of DNA [18]. For example, alterations in the methylation status of CpG islands in the promoter regions of genes implicated in obesity, appetite control, and metabolism have been shown to occur in DNA isolated from blood and breast tissue of obese compared to lean individuals [19–22]. Tobacco smoking, meanwhile, has been associated with alterations in DNA methylation of multiple cancer-related genes in studies focused on single candidate genes as well as in genome-wide methylation studies of prostate cancer, the bronchial epithelium, and peripheral blood mononuclear cells [23–26].

These observations led us to use HumanMethylation450 (HM450) BeadChips to evaluate epigenome-wide patterns of DNA methylation in a collection of human esophageal tissue samples, including cases of Barrett’s esophagus (BE), Barrett’s with low- or high-grade dysplasia, and esophageal adenocarcinoma (EAC). We were interested in determining whether BMI, tobacco smoking, and/or gender were associated with increased or decreased DNA methylation at specific CpG dinucleotides or in particular genomic regions, which would support a possible functional role in the pathogenesis of EAC. We also focused on whether epigenetic alterations linked with these demographic features associated with particular molecular or cancer-related pathways in order to assess for possible mechanisms through which alterations in the DNA methylation status may be involved in the formation of BE and/or EAC.

## Results

### Differences in the methylation status of genes in obesity-related pathways are associated with BMI status

Obesity has been consistently associated with an increased risk for developing both BE and EAC, yet little is known about the mechanisms involved in this elevated risk [27, 28]. While it is likely that both somatic genetic and epigenetic alterations play a role in the pathogenesis of BE and EAC, there is currently very little information about the relationship between Barrett’s esophagus and esophageal adenocarcinoma, obesity, and aberrant DNA methylation. From the 81 samples we analyzed on the HM450 array, body mass index (BMI) data were available for 46 cases, including 15 BE, 14 LGD, nine HGD, and eight EAC cases. We classified each of these samples as arising in the setting of either high BMI (BMI > 30) or low BMI (BMI ≤ 30). For female patients (*N* = 7), there were three in the low BMI and four in the high BMI groups. First, we determined whether the BE samples from the high BMI group (*N* = 11) had global DNA methylation alterations that were more closely related to HGD and/or EAC cases compared to the BE samples from study subjects with low BMI (*N* = 4). We found that high and low BMI BE cases tend to cluster together and that the high BMI BE cases did not appear to be more related to HGD/EAC than the low BMI BE cases (data not shown).

**Fig. 1:**
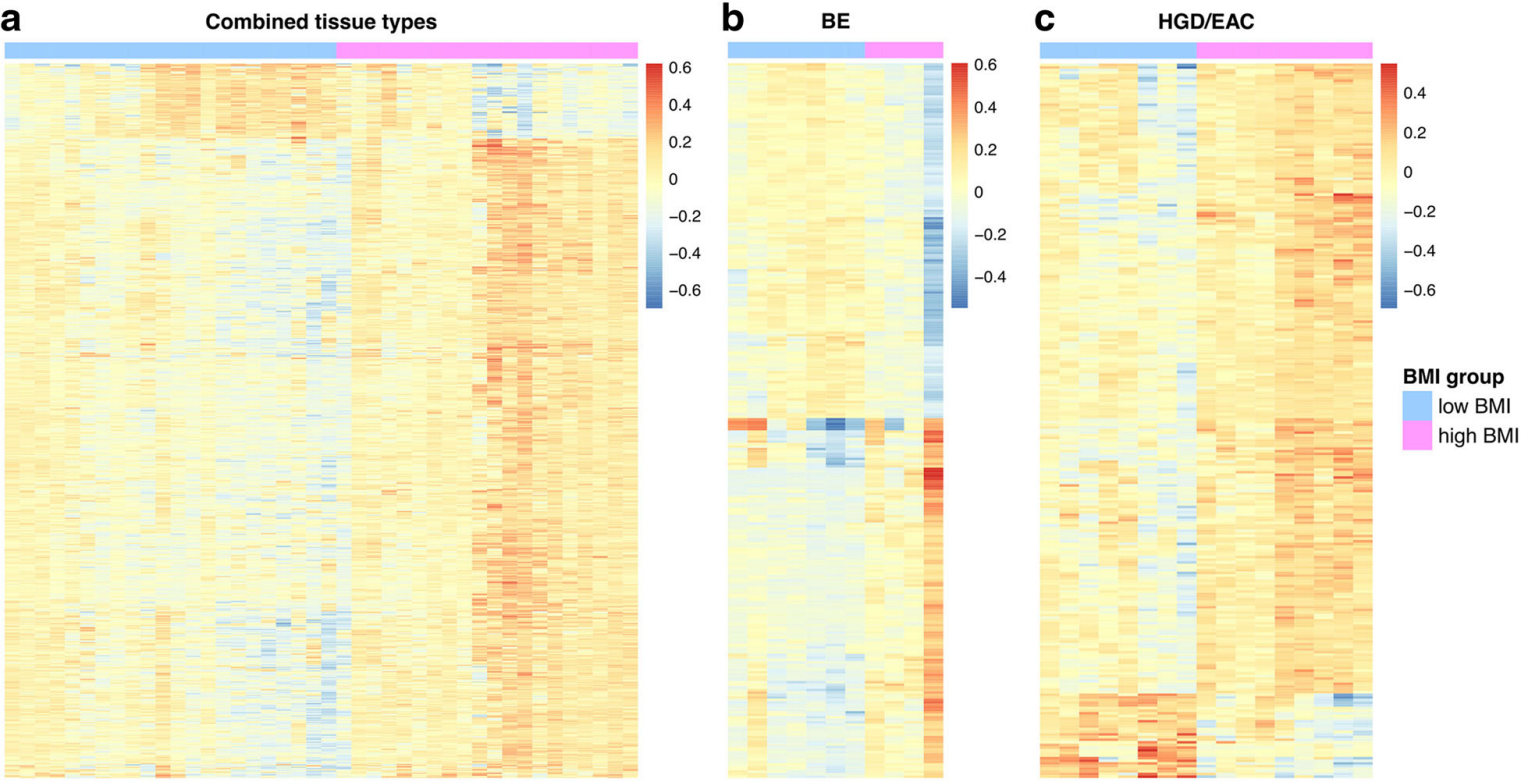
Dendrograms depicting DML when comparing high to low BMI cases. Because absolute differences in methylation (i.e., beta values) between cases were small, these heatmaps illustrate relative differences in methylation between cases instead of absolute beta values. **a** High vs. low BMI, all cases (BE, LGD, HGD, and EAC) combined. **b** High vs. low BMI, BE cases. **c** High vs. low BMI, HGD/EAC cases

Next, we assessed for differentially methylated loci (DML) that varied between the combined esophageal tissue samples (BE, LGD, HGD, EAC) from individuals with high vs. low BMI. Using criteria for DML of a *p* value <0.001 and *Δβ* between high BMI and low BMI > 0.10, we found a total of 974 DML between the high and low BMI groups, including 226, 471, and 277 DML located in promoter, intragenic, and intergenic regions, respectively. A dendrogram depicting the DML between high and low BMI patients is shown in Fig. 1. One hundred and eighty-two (182) DML were located in CpG islands and 376 were located in CpG island shores (within 2 kb of a transcription start site [29]). We also found 352 DML (36.1 % of the total 974 DML) that were cancer associated, which we defined as loci that were differentially methylated between the normal squamous (SQ; *N* = 12) and EAC (*N* = 24) cases on the HM450 array. In general, the high BMI cases showed increased methylation at the DML, with 872 out of 974 DML (89.5 %) demonstrating elevated methylation in high vs. low BMI cases. The DML with the greatest statistical significance (*p* < 5 × 10^−6^) associated with BMI are shown in Table 1.

**Table 1:** Differentially methylated loci (*p* < 5 × 10^−6^): high vs. low BMI cases (BE, LGD, HGD/EAC combined)

Probe ID	Gene	Average β low BMI	Average β high BMI (overall change)	*p* value	Probe location	Relation to CpG island	Relative expression BE vs. normal^	Relative expression EAC vs. normal^	Cancer associated?
cg11839020	*LRRC8D*	0.29	0.41 (↑)	4.06E-09	5′UTR	Shore	1.59–1.78*	1.44–1.86*	N
cg11027822	*ITGA6*	0.62	0.72 (↑)	1.97E-07	Body	Open sea	1.57–3.96*	1.48–2.66*	Y
cg25872281	*TMUB1*	0.66	0.81 (↑)	3.56E-07	TSS1500; body	Island	1.32*	NS	N
cg22984132	*TMUB1*	0.51	0.62 (↑)	3.64E-07	TSS1500; body	Island	1.32*	NS	N
cg26314478	*ESPNP*	0.49	0.60 (↑)	3.86E-07	Body	Shelf	NS	NS	N
cg09458237	*HSPA12B*	0.51	0.66 (↑)	4.46E-07	TSS1500	Shore	1.37*	1.89–6.50*	Y
cg06393286	*FAM43B*	0.48	0.36 (↓)	4.64E-07	1st exon	Island	NS	NS	Y
cg09058554	*SLC25A33*	0.39	0.51 (↑)	6.40E-07	Body	Shore	NS	1.23*	Y
cg14950321	*PLIN5*	0.31	0.42 (↑)	7.21E-07	Body	Shore	4.17*	NS	N
cg02134660	*FAM83B*	0.52	0.72 (↑)	1.17E-06	TSS1500	Shore	NS	NS	N
cg25302888	*TMUB1*	0.65	0.77 (↑)	1.23E-06	TSS1500; body	Island	1.32*	NS	N
cg05137975	*C6orf168*	0.53	0.70 (↑)	1.52E-06	Body	Open sea	NS	3.64*	N
cg16957569	*IDO2*	0.56	0.69 (↑)	1.68E-06	TSS1500	Open sea	NS	NS	N
cg00831127	*EPHB2*	0.26	0.49 (↑)	2.33E-06	Body	Shore	1.62–6.30*	1.40–7.83*	Y
cg25229964	*CNKSR1*	0.59	0.71 (↑)	2.39E-06	TSS1500	Open sea	NS	NS	N
cg19513232	*CAMK2A*	0.39	0.52 (↑)	2.43E-06	Body	Open sea	NS	NS	N
cg10976975	*BMP10*	0.72	0.83 (↑)	2.53E-06	5′UTR; 1st exon	Open sea	NS	NS	N
cg04025965	*TMUB1*	0.58	0.72 (↑)	2.66E-06	TSS1500; body	Island	1.32*	NS	N
cg02233614	*PFKFB2*	0.29	0.39 (↑)	2.82E-06	5′UTR	Shore	1.32*	1.12*	N
cg06020352	*IRF8*	0.36	0.50 (↑)	2.92E-06	TSS1500	Shore	1.80–6.36*	3.85–7.56*	N
cg08526705	*MYC*	0.61	0.74 (↑)	2.99E-06	Body	Shore	NS	3.89*	N
cg08943714	*HECA*	0.28	0.42 (↑)	3.66E-06	Body	Open sea	NS	NS	N
cg17161520	*TBC1D10C*	0.46	0.57 (↑)	3.92E-06	Body	Shelf	NS	1.14*	N
cg10583322	*MEGF11*	0.49	0.65 (↑)	4.15E-06	Body	Open sea	NS	1.69*	Y
cg02059867	*RAPGEFL1*	0.42	0.57 (↑)	4.50E-06	1st exon; 5′UTR	Island	NS	NS	N

*5′UTR* = 5′ untranslated region; *TSS1500* = 1500 bp’s upstream from transcription start site; *Shore* = DNA sequence up to 2 kb from CpG island; *Shelf* = DNA sequence 2–4 kb from CpG island; Open sea = DNA sequence >4 kb from CpG island; *NS* = not significant; ^ = relative gene expression data obtained from www.oncomine.org; *p ≤ 0.05; (↑)/(↓) = increase/decrease in methylation in high BMI group vs. the low BMI group

**Fig. 2:**
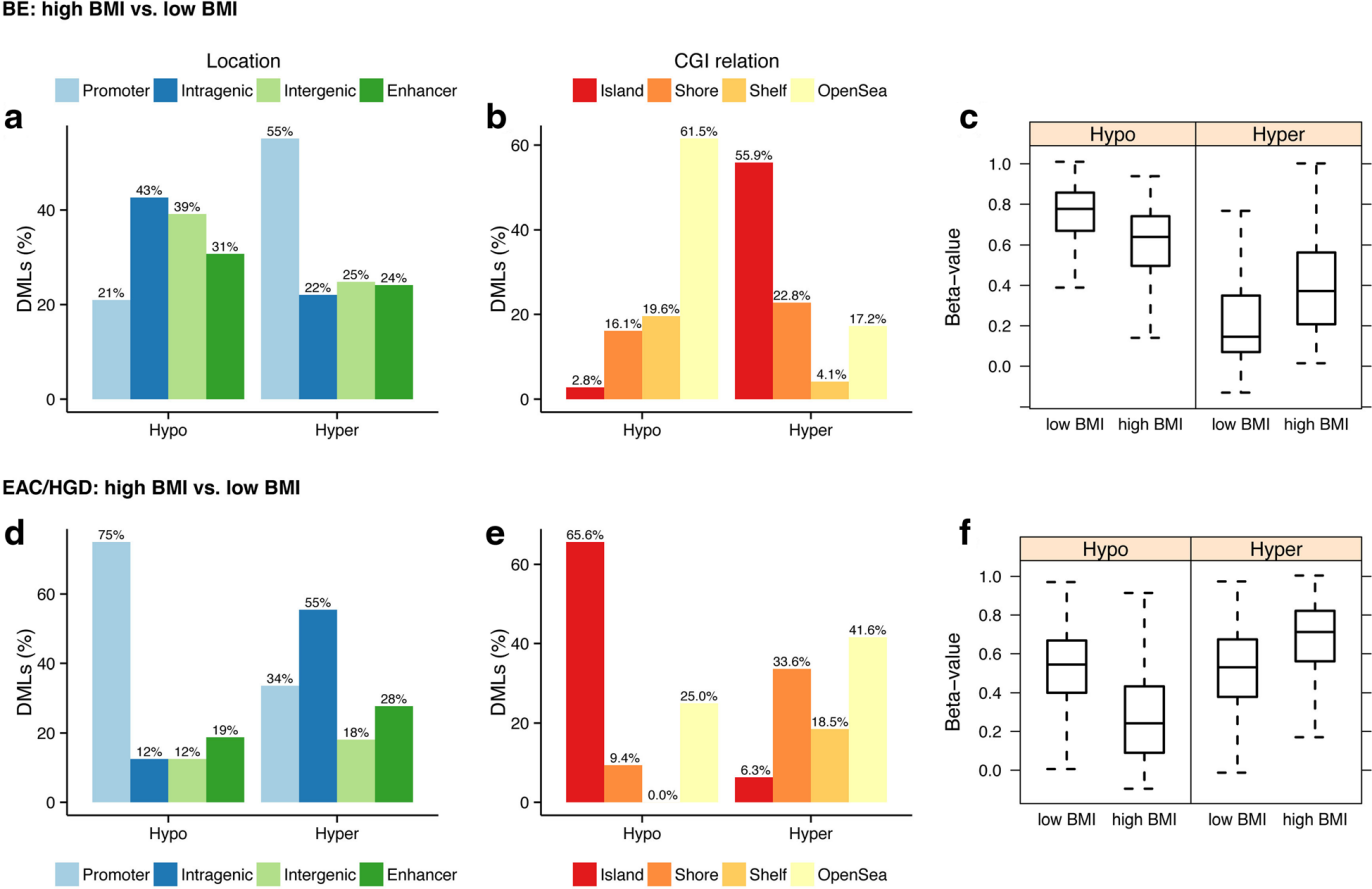
Genomic location, relationship to CpG islands, and methylation status of DML when comparing high vs. low BMI esophageal samples. In each panel, “Hypo” refers to percentage of DML that are hypomethylated in high BMI vs. low BMI samples; “Hyper” refers to percentage of DML that are hypermethylated in high BMI vs. low BMI samples. On the Y axis, DMLs (%) refers to the percentage of the total DML that are associated with a particular genomic location (**a**, **d**) or CGI relationship (**b**, **e**). Percentages may add up to more than 100 % because some probes were classified with more than one designation. Beta values are equivalent to percent methylation. **a** DML when comparing high BMI to low BMI BE cases by genomic region. Non-promoter regions were enriched with hypomethylated loci (p = 0.008), whereas promoter regions were borderline-enriched with hypermethylated loci (*p* = 0.06). **b** Location of DML when comparing high BMI to low BMI BE cases with respect to CpG island location. Non-CGI regions were enriched with hypomethylated loci (*p* = 8.4 × 10^−8^), whereas CpG island regions were enriched with hypermethylated loci (*p* = 0016). **c** Box and whisker plots showing distribution of DML that are hypomethylated in the high vs. low BMI BE cases (*left*) and hypermethylated in the high vs. low BMI BE cases (*right*). **d** DML when comparing high BMI to low BMI HGD/EAC cases by genomic region. Promoter regions were enriched with hypomethylated loci (*p* = 2.7 × 10^−6^). **e** Location of DML when comparing high BMI to low BMI HGD/EAC cases with respect to CpG island location. CpG island regions were enriched with hypomethylated loci (1.9 × 10^−6^), whereas non-island regions were enriched with hypermethylated loci (*p* = 6.5 × 10^−5^). **f** Box and whisker plots showing distribution of DML that are hypomethylated in the high vs. low BMI HGD/EAC cases (*left*) and hypermethylated in the high vs. low BMI HGD/EAC cases (*right*)

We also evaluated the association of BMI with tissue DNA methylation in the separate histologic types of esophageal tissues (e.g., BE, BE with LGD, BE with HGD, and EAC). We compared methylation in the high BMI (*N* = 4) vs. low BMI (*N* = 11) BE cases, the high BMI (N = 7) vs. low BMI (N = 7) LGD cases, and the high BMI (N = 9) vs. low BMI (N = 8) HGD/EAC cases. Table 2 summarizes the DML found when comparing these groups. The methylation status of the high compared to low BMI BE cases with respect to genomic regions and CpG island location is shown in Fig. 2. In general, in the BE cases, DML located in promoters and CpG islands were hypermethylated in high BMI vs. low BMI cases, whereas DML located elsewhere were hypomethylated in high BMI vs. low BMI cases. In contrast to this, DML in the HGD/EAC cases were hypermethylated at all functional regions as well as CpG island shores, shelves [30], and open seas in the high BMI vs. low BMI cases but not at CpG islands (Fig. 2).

**Table 2:** Differentially methylated loci: high BMI vs. low BMI cases separated by tissue type

Tissue	Total number of DML	Promoter DML (%)	Intragenic DML (%)	Intergenic DML (%)	CpG island DML (%)	CpG shore DML (%)	Cancer-associated DML (%)
BE	288	85 (29 %)	129 (45 %)	74 (26 %)	113 (39 %)	108 (37 %)	60 (21 %)
LGD	372	120 (32 %)	166 (45 %)	86 (23 %)	226 (61 %)	166 (45 %)	252 (68 %)
HGD/EAC	270	73 (27 %)	156 (58 %)	41 (15 %)	53 (20 %)	111 (41 %)	40 (15 %)

DML defined by *p* value <0.001 and *Δβ* value (high BMI vs. low BMI) > 0.10 while controlling for age

We also looked to see if any of the DML between the high and low BMI BE cases overlapped with any of the DML when comparing BE to EAC, in order to determine if methylation alterations in obese individuals with BE might be associated with progression to HGD/EAC. We did find nine probes that overlapped between these groups, including those targeting the genes *HLA-DPA1*, *TBR1*, *OSR2*, *TMEM63A*, *CD300E*, and *UBD*/*FAT10. UBD*/*FAT10*, which we found to be hypomethylated in high BMI BE patients, is of interest as this gene has been shown to be overexpressed in hepatocellular carcinoma (HCC) and is thought to modulate the β-catenin/TCF4 pathway and drive HCC invasion and metastasis [31].

Because of the potential for DNA methylation alterations to modify gene expression, we next assessed the methylation status of CpGs located in genes associated with signaling pathways and biological mediators implicated in obesity-associated cancers [17, 32, 33] in the esophageal tissues from the subjects with low vs. high BMI. With regard to the insulin and insulin growth factor 1 (IGF-1) related pathways, we observed increased methylation of *IGFBP1* (average beta = 0.11 in low BMI cases and 0.27 in high BMI cases) and *IRS2* (average beta = 0.11 in low BMI cases and 0.36 in high BMI cases) in the high BMI compared to low BMI BE cases. Both genes were hypermethylated in the high BMI cases in a CpG island located within exon 1. Unlike with BE cases, genes of the insulin or IGF-1 pathways did not show altered methylation in high vs. low BMI cases in the LGD, HGD, or EAC tissue sets. We also examined molecular pathways associated with adipose inflammation, which has been shown to mediate obesity-related cancer [32] and found the proinflammatory gene IL-1β (*IL1B*) to be hypermethylated in high vs. low BMI cases when we assessed the combined esophageal tissue sets. We also found hypermethylation of *IL1B* in the HGD/EAC cases from high BMI subjects. For the combined cases, the average beta was 0.25 (SD = 0.10, 95 % CI = 0.21–0.30) in low BMI cases and 0.35 (SD = 0.12, 95 % CI = 0.30–0.41) in high BMI cases and for the HGD/EAC cases, average beta was 0.20 (SD = 0.08, 95 % CI = 0.12–0.27) in low BMI cases and 0.38 (SD = 0.11, 95 % CI = 0.30–0.47) in high BMI cases. Of interest, adiponectin and leptin have also been implicated in obesity-associated cancer [34, 35]; however, we did not observe any differences in the DNA methylation status of genes involved in leptin or adiponectin pathways in any of the esophageal tissue sets in the high vs. low BMI subjects.

### There are numerous differentially methylated regions (DMR) between individuals with high and low BMI in esophageal tissues

**Fig. 3:**
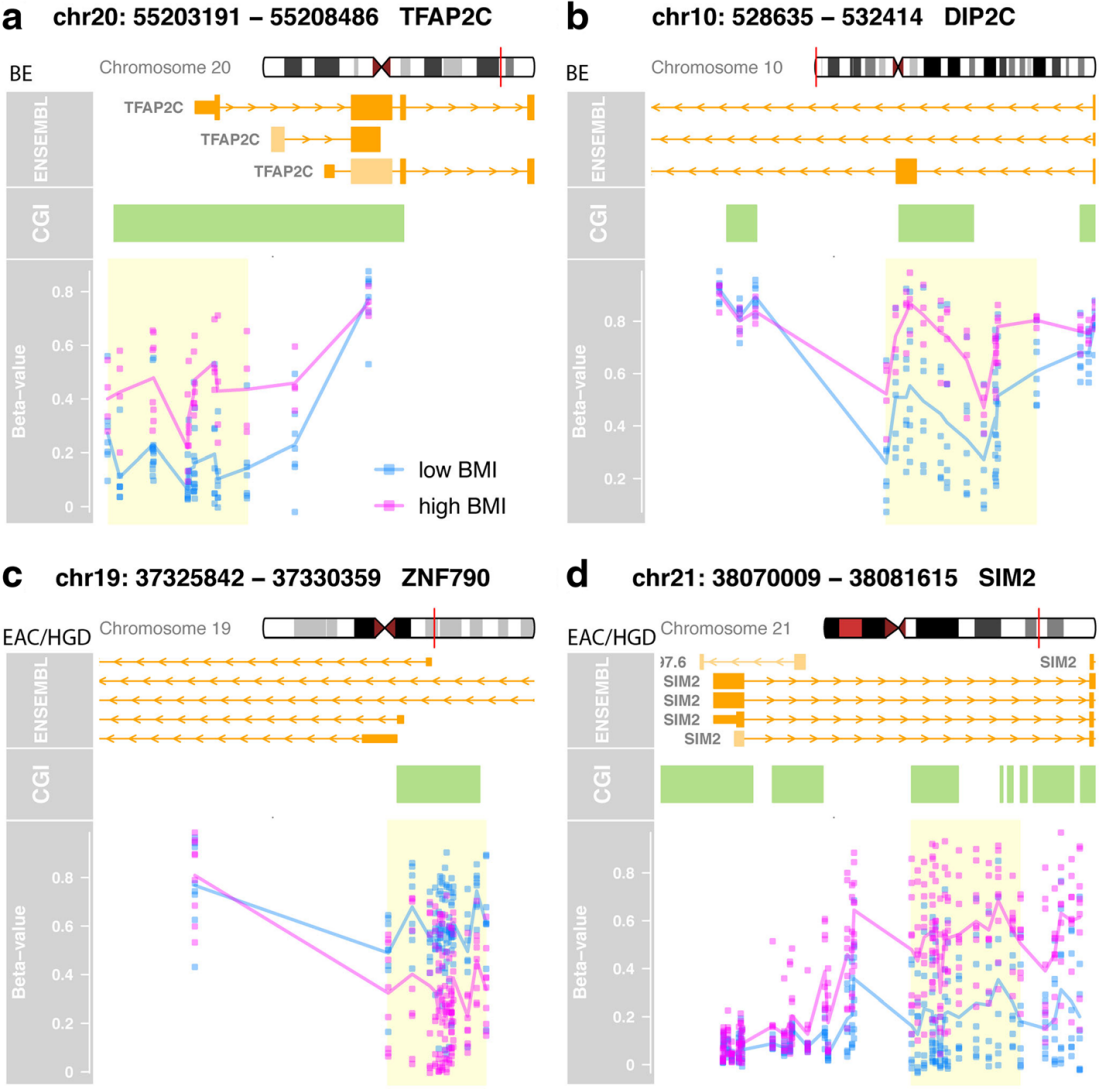
Selected genes containing differentially methylated regions (DMR) when comparing tissue samples from subjects with high vs. low BMI. The presence of concordant aberrant methylation is seen in the contiguous CpG sites in these DMR. Each panel contains the gene name and chromosomal location, alternate transcripts, exons and introns (*large* and *small orange boxes*), location of CpG islands (*green boxes*), DML (*blue* and *pink dots*), and the DMR (*yellow box*). Cases with BMI ≥30 are shown in *pink* and BMI < 30 in *blue*. **a** *TFAP2C* gene, BE cases. **b** *DIP2C* gene, BE cases. **c** *ZNF790*, HGD/EAC cases. **d** *SIM2* gene, HGD/EAC cases

The analysis described above was focused on the methylation status of individual CpG dinucleotides located in promoters, gene bodies, and intergenic regions. In light of recent studies suggesting gene expression changes are highly correlated with the aberrant methylation of large regions of DNA, called differentially methylated regions (genomic areas where numerous contiguous CpGs demonstrate significant concordant methylation alterations) [36, 37], we next assessed for differentially methylated regions (DMR) in the esophageal tissue samples from the low vs. high BMI subjects. Among the BE cases, there were DMR in 10 genes that differed between the high and low BMI groups (FWER < 0.10, *Δβ* > 0.10, and at least two contiguous CpG dinucleotides differentially methylated). Examples of two of these genes, *TFAP2C* and *DIP2C*, are shown in Fig. 3. Among the HGD/EAC cases, 31 DMR were identified using the same criteria, including regions in the genes *ZNF790* and *SIM2* (Fig. 3). We did not find any DMR within prominent genes in the insulin, IGF-1, TNF-α, or leptin pathways.

### A comparison of genes showing differential methylation between high vs. low BMI cases demonstrates the involvement of cancer-related pathways and gene sets

We used the NCI Pathway Interaction Database (NCI-PID), Kyoto Encyclopedia of Genes and Genomes (KEGG) database, and the list of Gene Ontology (GO) terms to identify biological processes or pathways that were over- or under-represented based on genes containing DML between the esophageal tissue sets in the subjects with either high or low BMI status. As mentioned previously, we defined “cancer-associated” probes as those that were differentially methylated between EAC and SQ cases on the array.

Among the BE cases, we found one NCI-PID pathway, “direct p53 effectors”, which includes the differentially methylated gene *RDX* from our dataset, associated with methylation differences between high and low BMI groups. There were 13 KEGG pathways (including “cell adhesion molecules”) and 77 GO terms (including “response to growth hormone” and “biological adhesion”) that were represented in the differentially methylated genes in the BE samples from the high vs. low BMI subjects. The list of GO terms is shown in Additional file 1: Table S2.

With respect to the HGD/EAC cases, there were no NCI-PID pathways that were significantly associated with methylation differences between high and low BMI status after restricting our analysis to only cancer-related genes. There was one KEGG pathway (“Wnt signaling”) and 87 GO terms (such as “tissue morphogenesis” and “response to TGF-beta”) differentially methylated between HGD/EAC cases from subjects with high BMI vs. low BMI (*p* value <0.05) (Additional file 2: Table S3).

### Gender-related differences in DNA methylation in esophageal tissues

Little is known about gender-specific variations in DNA methylation in most tissues, including the esophagus. Previous studies have shown that repetitive elements and specific CpG dinucleotides isolated from blood samples demonstrate modestly increased methylation in males compared to females [38, 39]. Another study of four candidate genes in colorectal adenocarcinoma cells demonstrated that males had increased methylation of *MTHFR*, *CALCA*, and *MGMT* compared to females [40].

To the best of our knowledge, a genome-wide analysis of gender differences in DNA methylation in the esophagus has not been previously reported. Using HM450 array analysis of BE, HGD, and EAC esophageal samples from 118 males and 23 females, we found numerous CpG sites that were differentially methylated between the genders after excluding probes on the X and Y chromosomes and after accounting for differences in the age between the men and women in our study. When we combined the BE, HGD, and EAC cases, there were 1092 DML, including 369, 421, and 402 DML located in promoter, intragenic, and intergenic regions, respectively. From this list, there were 402 DML where the mean beta value difference between males and females was >0.10 and *p* value was <0.001. These DML were associated with CpGs in genes such as *DUSP22*, a regulator of estrogen receptor alpha mediated signaling, *FRG1B*, which is involved in pre-mRNA splicing, and *CGREF1*, which mediates cell-cell adhesion in a calcium-dependent manner. Of these 402 DML, 327 (81.3 %) were more highly methylated in females. The DML with the greatest statistical significance (*p* < 5 × 10^−6^) between males and females are listed in Table 3. Of interest, half of the top DML were located in CpG islands.

**Table 3:** Differentially methylated loci (*p* < 5 × 10^−6^): females vs. males (BE, HGD/EAC combined)

Probe ID	Gene	Average β females	Average β males (overall change)	*p* value	Probe location	Relative expression BE vs. normal^	Relative expression EAC vs. normal^	Relation to island
cg17272795	*ZNF37A*	0.34	0.10 (↓)	1.76E-09	TSS1500	1.06*	−1.05*	Shore
cg07753967	*FRG1B*	0.44	0.10 (↓)	2.13E-09	TSS1500	NS	NS	Island
cg25791279	*PISD*	0.26	0.15 (↓)	8.77E-09	TSS200	NS	2.17–3.20*	Shore
cg25489030	*FRG1B*	0.46	0.30 (↓)	3.63E-08	Body	NS	NS	Island
cg02531214	*ZNF37A*	0.34	0.13 (↓)	1.76E-07	TSS1500	1.06*	−1.05*	Shore
cg20811988	*FRG1B*	0.33	0.11 (↓)	7.17E-07	Body	NS	NS	Island
cg03395511	*DUSP22*	0.11	0.42 (↑)	8.15E-07	TSS200	NS	NS	Shore
cg14815891	*FRG1B*	0.33	0.08 (↓)	1.03E-06	Body	NS	NS	Island
cg01516881	*DUSP22*	0.07	0.23 (↑)	1.23E-06	Body	NS	NS	Island
cg21508714	*RBM20*	0.31	0.12 (↓)	1.42E-06	Body	NS	NS	Island
cg11386792	*RBM20*	0.44	0.13 (↓)	1.74E-06	Body	NS	NS	Island
cg16004008	*NRGN*	0.35	0.20 (↓)	1.91E-06	TSS1500	1.45–1.46*	1.57–3.23*	Shore
cg03066577	*C3orf55*	0.26	0.11 (↓)	2.17E-06	5′UTR; 1st exon; body	NS	4.95*	Island
cg21548813	*DUSP22*	0.08	0.36 (↑)	2.25E-06	TSS1500	NS	NS	Shore
cg14819088	*SLC34A1*	0.67	0.78 (↑)	2.52E-06	TSS200	NS	NS	Open sea
cg15383120	*DUSP22*	0.08	0.38 (↑)	2.52E-06	TSS200	NS	NS	Shore
cg18110333	*DUSP22*	0.09	0.39 (↑)	3.92E-06	1st exon; 5′UTR	NS	NS	Island
cg16602806	*MTRR*	0.19	0.09 (↓)	4.59E-06	Body; TSS1500; 5′UTR	1.65*	1.77*	Shore
cg25959506	*RASD2*	0.27	0.11 (↓)	4.91E-06	TSS1500	NS	NS	Island

*5′UTR* = 5′ untranslated region; *TSS200/TSS1500* = 200 or 1500 bp’s upstream from transcription start site; *Shore* = DNA sequence up to 2 kb from CpG island; *Open sea* = DNA sequence >4 kb from CpG island; *NS* = not significant; ^ = relative gene expression data obtained from www.oncomine.org; *p ≤ 0.05; (↑)/(↓) = increase/decrease in methylation in male vs. females

### Tobacco use is associated with DNA hypermethylation in the esophagus

Tobacco smoking, which is a well-known risk factor for Barrett’s esophagus and EAC, has been associated with alterations in DNA methylation in peripheral blood lymphocytes [26, 41]. However, little is known about the relationship between smoking and DNA methylation alterations in esophageal tissues, including BE and EAC. To investigate this further, we assessed the relationship between smoking and aberrant DNA methylation in samples from subjects for which we had data on tobacco use. We divided cases into “smokers” (which included both current and former smokers) and “nonsmokers;” we did not further segregate smokers by current smoking status, pack-years, etc. due to the relatively small number of cases available. We first compared BE nonsmokers (*N* = 7) to BE smokers (*N* = 9) using principal component analysis (PCA) to determine whether methylation patterns of BE smokers more closely resembled the patterns we observed in LGD, HGD, and/or EAC cases compared to BE nonsmokers. When we examined the 1000 loci with the most variable methylation between groups, we did not find the BE smokers were grouped more closely with LGD/HGD/EAC than BE nonsmokers (data not shown).

**Fig. 4:**
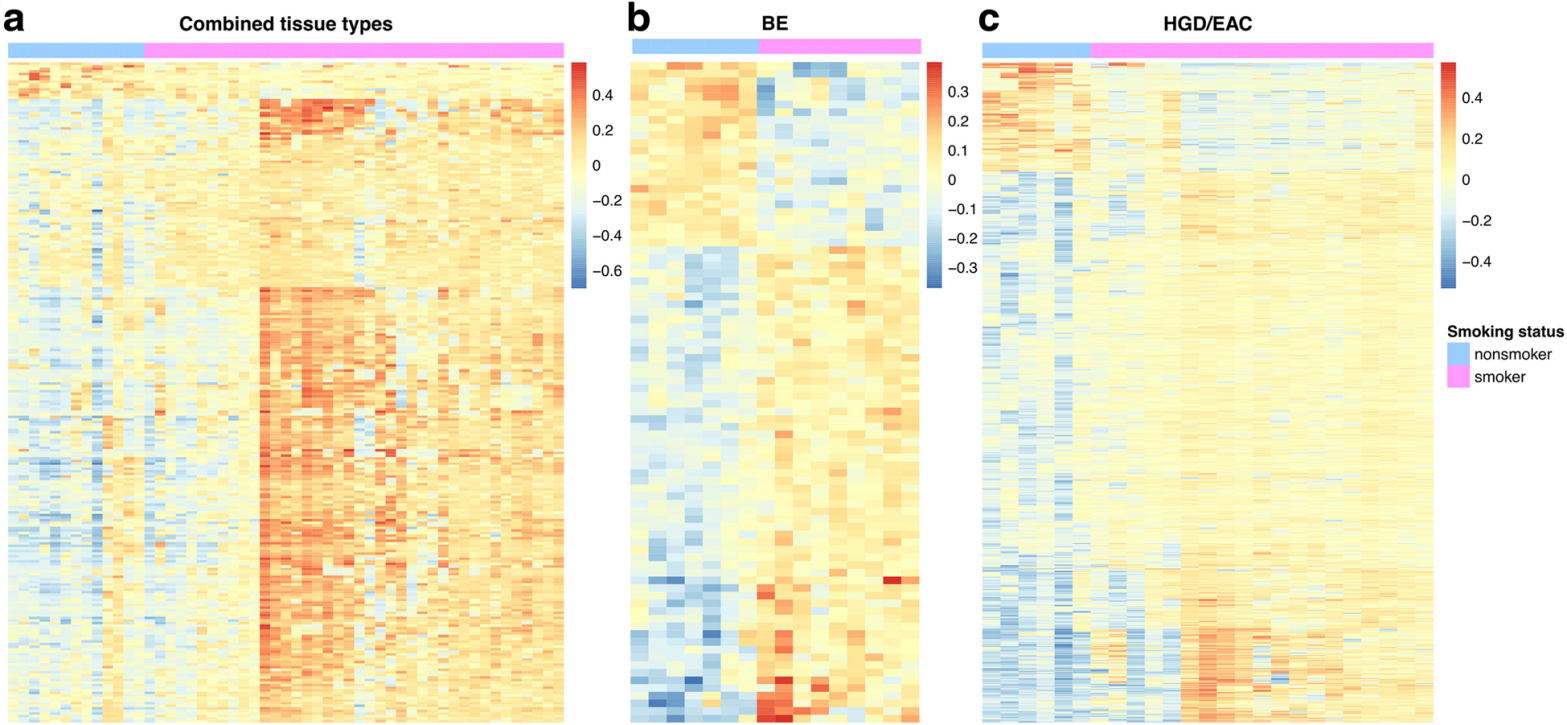
Dendrograms depicting DML when comparing smokers and nonsmokers. Because absolute differences in methylation (i.e., beta values) between cases were small, these heatmaps illustrate relative differences in methylation between cases instead of absolute beta values. **a** smokers vs. nonsmokers, all cases (BE, LGD, HGD, and EAC) combined. **b** Smokers vs. nonsmokers, BE cases. **c** Smokers vs. nonsmokers, HGD/EAC cases

Next, we evaluated 54 esophageal samples of various histologic types (BE, LGD, HGD, and EAC) for global alterations in DNA methylation associated with tobacco smoking. After controlling for differences associated with the histological diagnosis (BE, LGD, or HGD/EAC), we found a total of 256 DML between the smokers (*N* = 40) and nonsmokers (*N* = 14) (*Δβ* > 0.10, *p* < 0.001). Heatmaps depicting the DML between smokers and nonsmokers are shown in Fig. 4.

These DML included 98, 40, and 118 loci located in promoter, intragenic, and intergenic regions, respectively. Two hundred forty-two (242) of the 256 DML (94.5 %) were more highly methylated in smokers compared to nonsmokers, and 105 of the 256 DML (41.0 %) affected cancer-associated genes, as based on the criteria described above. The DML with the greatest statistical significance (*p* < 1 × 10^−4^) associated with smoking are shown in Table 4.

**Table 4:** Differentially methylated loci (*p* < 1 × 10^−4^): ever smokers vs. never smokers (BE, LGD, HGD/EAC combined)

Probe ID	Gene	Average β ever smoker	Average β never smoker (overall change)	*p* value	Probe location	Relation to island	Relative expression BE vs. normal^	Relative expression EAC vs. normal^	Cancer associated?
cg05951860	*CTTNBP2*	0.49	0.15 (↓)	5.80E-07	Body	Island	NS	NS	Y
cg15310873	*C20orf85*	0.74	0.60 (↓)	5.06E-06	Body	Island	NS	3.34*	N
cg23039279	*PROK2*	0.48	0.21 (↓)	1.92E-05	5′UTR; 1st exon	Island	NS	1.90*	Y
cg16024318	*SLC6A7*	0.39	0.20 (↓)	2.68E-05	5′UTR; 1st exon	Island	1.31*	1.17*	Y
cg20285514	*GNG4*	0.52	0.19 (↓)	2.82E-05	TSS200; 5′UTR	Island	NS	NS	Y
cg07657743	*WNT7A*	0.56	0.22 (↓)	3.93E-05	Body	Island	NS	NS	N
cg19169023	*TYRO3*	0.74	0.57 (↓)	4.24E-05	Body	Shore	NS	NS	N
cg25757598	*RALYL*	0.51	0.25 (↓)	4.59E-05	5′UTR; 1st exon	Island	1.16*	2.30*	N
cg14196840	*CTTNBP2*	0.37	0.11 (↓)	5.08E-05	Body	Island	NS	NS	Y
cg04842146	*RALYL*	0.46	0.15 (↓)	5.55E-05	TSS1500	Shore	1.16*	2.30*	Y
cg20620272	*C3orf50*	0.42	0.16 (↓)	5.77E-05	Body	Island	NS	NS	N
cg09374774	*FAM78A*	0.19	0.43 (↑)	5.80E-05	TSS200	Island	NS	1.04*	N
cg18016194	*RALYL*	0.50	0.29 (↓)	7.17E-05	5′UTR; 1st exon	Island	1.16*	2.30*	N
cg27603796	*CTTNBP2*	0.47	0.21 (↓)	7.21E-05	Body	Shore	NS	NS	Y
cg00818822	*RFX2*	0.90	0.80 (↓)	8.05E-05	Body	Island	1.24*	6.23*	N
cg10879116	*LPAR3*	0.51	0.26 (↓)	8.54E-05	5′UTR	Island	NS	NS	Y
cg11981631	*ABCC8*	0.59	0.30 (↓)	9.36E-05	Body	Island	1.38*	1.11–1.70*	Y
cg06600429	*GABRB2*	0.32	0.55 (↑)	9.75E-05	TSS1500	Shore	NS	NS	Y

*5′UTR* = 5′ untranslated region; *TSS200/TSS1500* = 200/1500 bp’s upstream from transcription start site; *Shore* = DNA sequence up to 2 kb from CpG island; *NS* = not significant; ^ = relative gene expression data obtained from www.oncomine.org; *p ≤ 0.05; (↑)/(↓) = increase/decrease in methylation never smokers vs. smokers

**Fig. 5:**
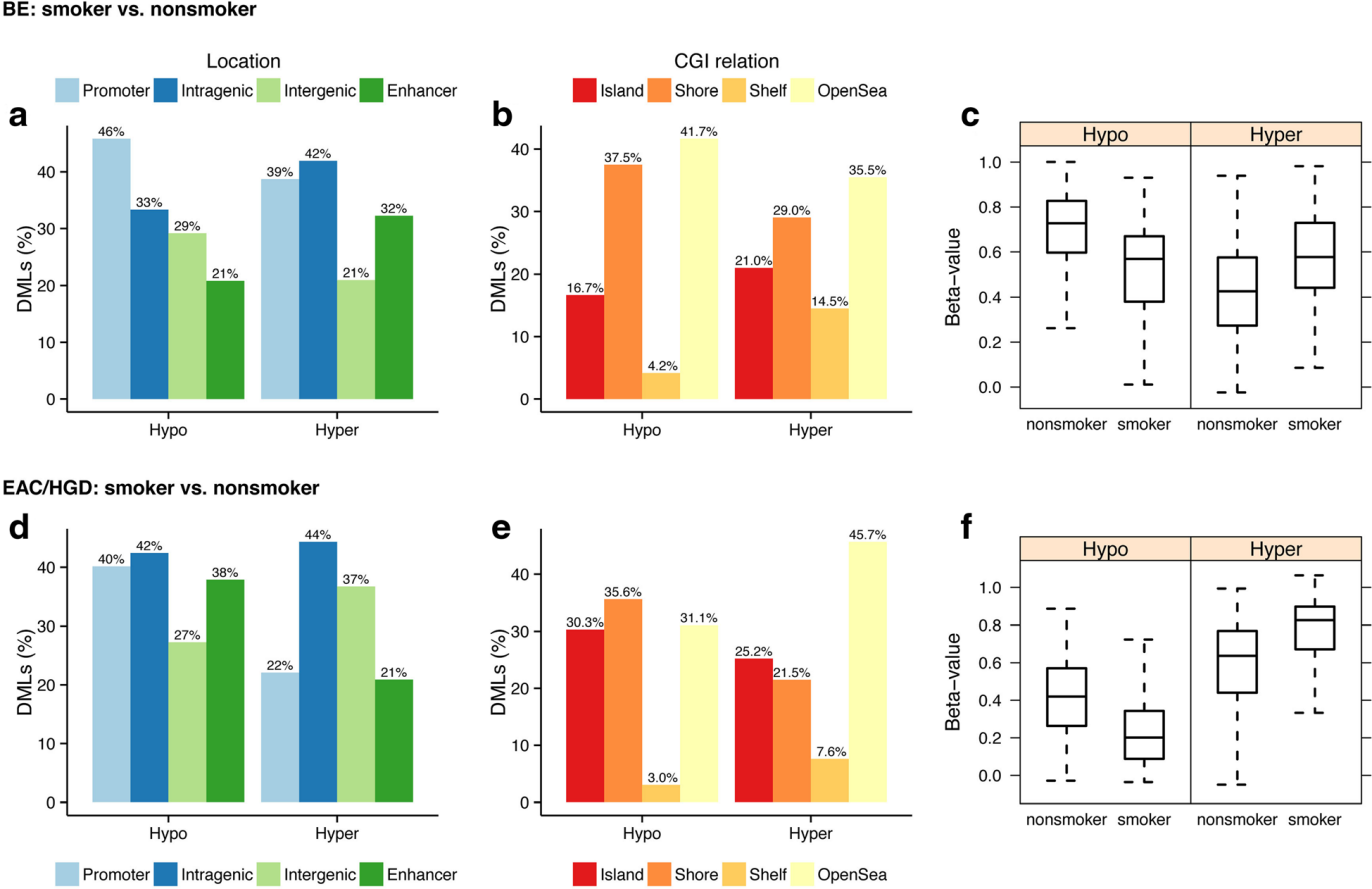
Genomic location, relationship to CpG islands, and methylation status of DML when comparing smokers and nonsmokers in esophageal samples. “Hypo” refers to percentage of DML that are hypomethylated in smokers vs. nonsmokers; “Hyper” refers to percentage of DML that are hypermethylated in smokers vs. nonsmokers. On the Y axis, DMLs (%) refers to the percentage of the total DML that are associated with a particular genomic location (**a**, **d**) or CGI relationship (**b**, **e**). Percentages may up to more than 100 % because some probes were classified with more than one designation. Beta values are equivalent to percent methylation. Note: for all regions, the distribution of hypo/hypermethylated DML compared to the expected distribution (based on all array probes) was not statistically significant. **a** DML when comparing smoker to nonsmoker BE cases by genomic region. **b** Location of DML when comparing smoker to nonsmoker BE cases with respect to CpG island location. **c** Box and whisker plots showing distribution of DML that are hypomethylated in the smoker vs. nonsmoker BE cases (*left*) and hypermethylated in the smoker vs. nonsmoker BE cases (*right*). **d** DML when comparing smoker vs. nonsmoker HGD/EAC cases by genomic region. **e** Location of DML when comparing smokers vs. nonsmoker HGD/EAC cases with respect to CpG island location. **f** Box and whisker plots showing distribution of DML that are hypomethylated in the smoker vs. nonsmoker BMI HGD/EAC cases (*left*) and hypermethylated in the smoker vs. nonsmoker HGD/EAC cases (*right*)

We also evaluated the association of tobacco use with DNA methylation in the separate esophageal tissue types (i.e., BE, LGD, HGD, and EAC). We assessed for DML in the BE smokers (*N* = 9) vs. BE nonsmokers (*N* = 7) and in the HGD/EAC smokers (*N* = 19) vs. HGD/EAC nonsmokers (*N* = 7) while controlling for age differences. We were not able to compare the LGD cases as all samples were from smokers. Table 5 summarizes the DML we identified for these comparisons and shows the functional genomic locations of the loci when comparing these groups. The methylation status of the BE and HGD/EAC tissues from smokers compared to nonsmokers with respect to genomic regions and CpG island location is shown in Fig. 5. In both BE and HGD/EAC cases, the DML from smokers showed much higher methylation in all genomic regions analyzed (Fig. 5).

**Table 5:** Differentially methylated loci: smokers vs. nonsmokers separated by tissue type

Tissue	Total No. DML	Promoter DML (%)	Intragenic DML (%)	Intergenic DML (%)	CpG island DML (%)	CpG shore DML (%)	Cancer-associated DML (%)
BE	86	25 (29 %)	43 (50 %)	18 (21 %)	23 (27 %)	28 (32 %)	21 (24 %)
HGD/EAC	802	152 (19 %)	417 (52 %)	233 (29 %)	279 (35 %)	395 (49 %)	79 (10 %)

DML defined by *p* value <0.001 and *Δβ* value (smoker vs. nonsmoker) > 0.10 while controlling for age

### There are numerous differentially methylated regions (DMR) in esophageal tissues based on tobacco use status

**Fig. 6:**
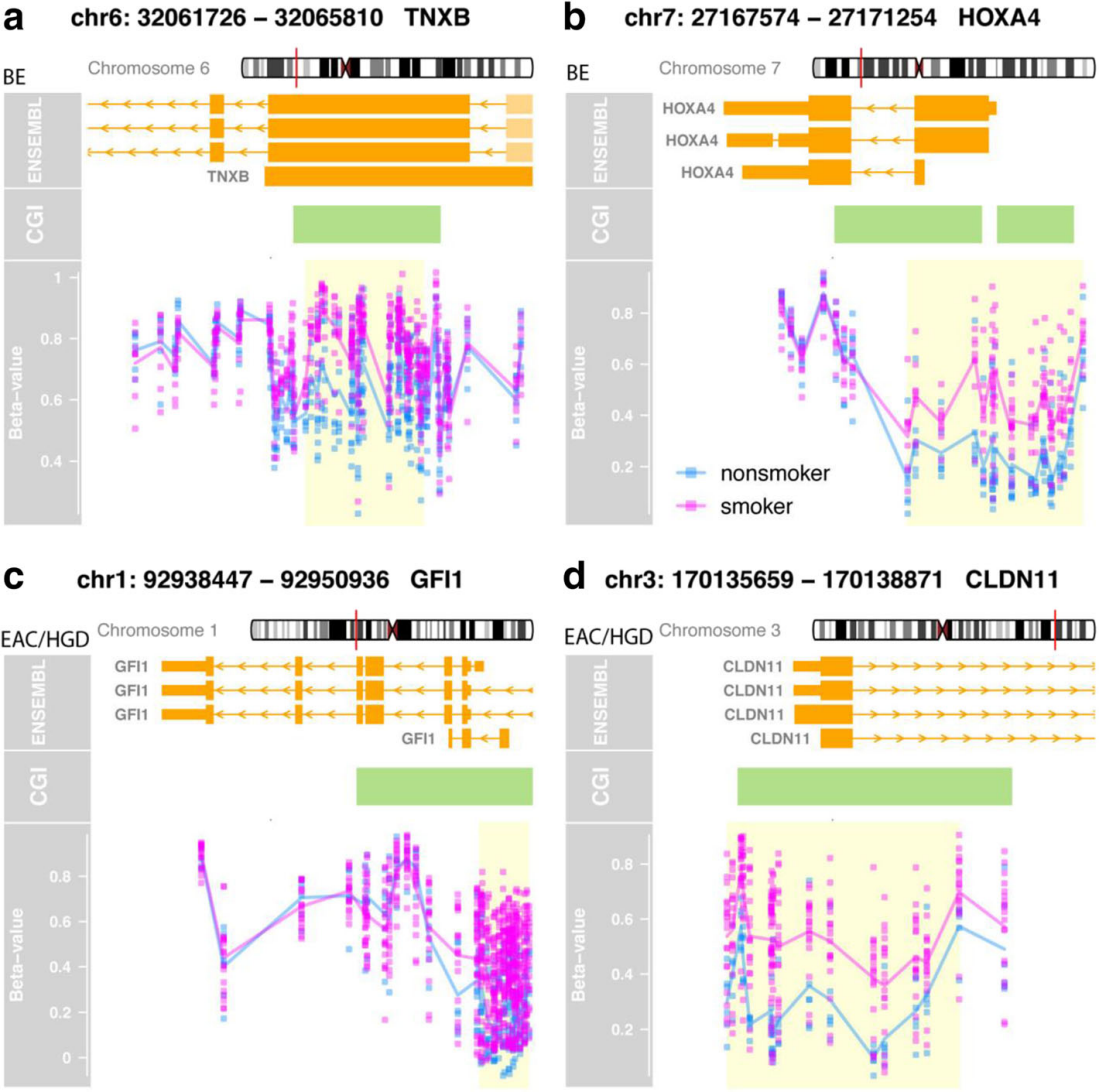
Selected genes containing differentially methylated regions (DMR) when comparing smoker and nonsmoker cases, depicting the location of multiple contiguous differentially methylated CpG sites. Each panel contains the gene name and chromosomal location, alternate transcripts, exons and introns (*large* and *small orange boxes*), location of CpG islands (*green boxes*), DML (*blue* and *pink dots*), and the DMR (*yellow box*). Smokers are shown in *pink* and nonsmokers in *blue*. **a** *TNXB* gene, BE cases. **b** *HOXA4* gene, BE cases. **c** *GFI1*, HGD/EAC cases. **d** *CLDN11* gene, HGD/EAC cases

As with the BMI-based comparison described above, we were interested in extending our analysis of differential DNA methylation between smokers and nonsmokers to include differentially methylated regions in addition to DML, which are single CpG sites. Among the BE cases, there were DMR found involving 13 genes when comparing smokers to nonsmokers (FWER < 0.10, *Δβ* > 0.10, and at least two contiguous CpG dinucleotides differentially methylated). These DMR were located within the genes *TNXB* and *HOXA4*, which are notable because TNXB is a member of the tenascin family and regulates cell-extracellular matrix interactions [42, 43] and *HOXA4* is a transcription factor previously shown to inhibit cell motility and to be aberrantly methylated in acute myeloid leukemia [44, 45] (Fig. 6). TNXB is normally more highly expressed in BE tissues compared to normal squamous esophagus (fold change = 3.39) whereas HOXA4 has not been shown to be differentially expressed in BE vs. normal esophagus (expression data obtained from www.oncomine.org). Among the HGD/EAC cases, we identified 29 DMR, including areas with altered methylation in the genes *GFI1*, which is a transcriptional repressor implicated in the regulation of p53 activity and Notch signaling [46, 47] and *CLDN11*, a cell adhesion protein involved in cell migration that is commonly altered in cancer [48] (Fig. 6). Normally, both GFI1 and CLDN11 have been shown to be more highly expressed in EAC tissues vs. normal esophagus (fold changes = 1.30 and 1.11–3.39, respectively; www.oncomine.org).

### Differences in esophageal DNA methylation between smokers and nonsmokers are associated with several cancer-related pathways and gene sets

We were interested to see which molecular and cancer-related pathways were associated with the epigenetic differences in the BE and EAC tissues from smokers as compared to nonsmokers. As with the BMI cases, we restricted our NCI Pathway Interaction Database (NCI-PID) analysis to only those DML that we considered to be “cancer related” to improve the likelihood these pathways would contain biologically plausible mechanisms involved in smoking-related BE and/or EAC formation.

Analysis of BE cases alone did not identify any NCI-PID pathways that were differentially methylated in BE smokers vs. nonsmokers. However, there was 1 KEGG pathway (“type 1 diabetes mellitus”) and 20 GO terms (including “positive regulation of mismatch repair” and “enteric smooth muscle cell differentiation”) that were differentially represented between the BE samples from smokers vs. nonsmokers (*p* < 0.05) (Additional file 3: Table S4).

When we compared DNA methylation in the HGD/EAC tissues of smokers and nonsmokers, we found two NCI-PID pathways associated with alterations in DNA methylation and smoking (FDR ≤ 0.05), including the “neurotrophic factor-mediated Trk receptor signaling” and “SHP2 signaling” pathways. The differentially methylated genes *NTRK2* and *NTRK3* were notable affected members of both of these pathways. There were no KEGG pathways but there were 217 GO terms (such as “localization of cell” and “regulation of cell migration”) that were differentially represented (Additional file 4: Table S5).

## Discussion

Genetic and epigenetic alterations are commonly found in BE and EAC and likely play a prominent role in driving the initiation and progression of BE to EAC. It is also well known that a variety of environmental factors associate with the risk of developing BE and/or EAC. Thus, we assessed the relationship between DNA methylation in the esophagus and known risk factors for BE and EAC using a genome-wide methylation platform. We also sought to describe the epigenetic differences between males and females in esophageal tissues in light of the known differences in BE and EAC incidence in men vs. women. With respect to demographic and behavioral variables, we were particularly interested in the correlation of BMI and tobacco use with DNA methylation since both are well-established risk factors for BE and EAC.

We assessed the methylation status of more than 485,000 CpG sites located in 99 % of the RefSeq genes in 81 esophageal tissues representative of the stages of esophageal adenocarcinoma development (BE, BE + LGD, BE + HGD, EAC). The annotation of array probes permitted us to determine whether differentially methylated loci were located in specific types of genomic regions (promoter, gene body, or intergenic) and to determine the relationship of differentially methylated loci (DML) to CpG islands (CpG island, shore, shelf, or open sea). Our analysis of the regions outside of promoter-related CpG islands is notable because an understanding of methylation alterations in areas with relatively low CpG density is becoming increasingly recognized to be important in diseases such as cancer [49, 50]. It has been shown that CpG-rich regions (i.e., CpG islands) demonstrate more stable DNA methylation across tissues and cell populations whereas methylation is more dynamic in CpG shores (within 2 kb of a CpG islands) and CpG shelves (within 4 kb of a CpG island). Furthermore, the methylation status of CpG shores and shelves appears to regulate gene expression [29, 51].

We initially investigated the relationship between DNA methylation and BMI in esophageal tissues. Elevated body mass index (BMI) is an established risk factor for BE and EAC, and we demonstrated that DNA isolated from individuals with BMI > 30 was differentially methylated at nearly 1000 CpG sites in combined BE, BE with low- and high-grade dysplasia, and EAC tissues when compared to samples from individuals with a low BMI status. Interestingly, nearly 90 % of the DML showed elevated methylation in the high BMI cases, and over 36 % of the total DML were cancer related. There were 20 % more cancer-related DML in the high BMI group than we would expect by chance alone since just 16 % of the total probes on the array are “cancer related” by our criteria as previously described. In the BE cases, DML located in promoters and CpG islands tended to be hypermethylated in those with high BMI which suggests a possible association between methylation and altered gene expression in those with elevated BMI as promoter hypermethylation has been associated with gene silencing [52]; this remains speculative given we did not have associated gene expression data. There was also evidence of altered methylation in BE and EAC samples from obese patients when we looked at differentially methylated regions (DMR), which are genomic regions that have multiple adjacent CpG sites showing concordant methylation changes. DMR are potentially more biologically important than differentially methylated individual CpG dinucleotides because they are indicative of larger scale epigenetic alterations that might be more relevant functionally [36, 53].

We were also interested in whether the high BMI BE cases displayed methylation alterations resembling the EAC cases, our rationale being these epigenetic alterations in the obese with BE might be markers for progression to dysplasia or cancer and provide some evidence of a biological role for the genes subjected to aberrant methylation. This was not the case, however, as the high BMI cases clustered more closely with the low BMI cases, not the EAC cases.

We subjected the DML to KEGG, Gene Ontology (GO), and NCI-PID analyses to determine whether particular molecular groups or pathways were associated with the methylation changes in obese individuals with BE, dysplastic BE, or EAC. Among the BE cases, we found epigenetic alterations in the direct p53 effectors pathway in individuals with elevated BMI. This included differentially methylated loci within the *RDX* gene, which encode a cytoskeletal component that has been shown to inhibit metastasis in gastric cancer [54].

*TP53*, the gene for p53, is a well-known tumor suppressor gene that is frequently lost early in BE through mutation or loss of heterozygosity (LOH) [55]. *TP53* LOH has been shown to identify a subset of BE patients who are at risk for progression to EAC [56, 57]. The finding of differential methylation involving the p53 pathway in BE from subjects with high vs. low BMI suggests a relationship between obesity and DNA methylation of cancer-related genes in the esophagus. Similar results have been found in other studies comparing methylation in obese to lean individuals. In a recent study of 345 breast cancer cases, the majority (87 %) of CpG sites analyzed showed elevated methylation in obese patients, particularly in estrogen receptor-positive tumors. Obesity was associated with the aberrant methylation of cancer-related genes involved with the immune response, cell growth, and DNA repair [22]. Several prior studies have compared DNA methylation in whole blood or peripheral blood leukocytes among obese and non-obese individuals [58–60]. In two of these studies, the gene *HIF3A* was found to be hypermethylated in the blood cells and adipose tissue of obese adults, suggesting perturbation of the hypoxia inducible transcription factor pathway in those with elevated BMI.

We were also interested in determining if there were genome-wide differences in esophageal DNA methylation between males and females. Previously, when we used a genome-wide approach to compare methylation in the normal colon between males and females, we found 82 DML between the groups, with females showing increased methylation at 69.5 % of the differentially methylated CpGs [61]. In the present study, we found 402 DML after controlling for age and histology, with 81.3 % showing higher methylation in females. Other studies have shown differences in autosomal DNA methylation by gender in the brain, saliva, and blood [9, 62, 63]. These results suggest that DNA methylation might function in the differentiation or maintenance of sexual dimorphism. An understanding of tissue-specific gender differences is also important in terms of understanding the role of environmental, behavioral, and demographic factors on alterations in DNA methylation in order to appropriately account for potentially confounding effects of gender [63].

Tobacco smoking is another known risk factor for developing Barrett’s esophagus and esophageal adenocarcinoma [64]. The mechanisms accounting for this risk are only partly understood and are believed to involve carcinogen-mediated mutations. Cigarette smoke contains multiple carcinogens which likely exert their effects via the induction of DNA adducts, aberrant DNA methylation and mutation, and chromosomal translocation [65, 66]. In order to define the association between tobacco use and aberrant DNA methylation in BE/EAC, we analyzed 54 esophageal samples of various histological types for global alterations in DNA methylation associated with tobacco smoking. We found 256 DML in these tissues between smokers and nonsmokers. Ninety-five percent (95 %) of these DML showed elevated methylation in the smoker group and 41.0 % were cancer related, which is 25 % more cancer-related DML than would be expected by chance alone.

The finding of widespread and frequent hypermethylation in BE, dysplastic BE, and EAC tissues of tobacco smokers suggests that tobacco-related epigenetic alterations may be a mechanism through which tobacco affects the development of BE and EAC. After enriching the DML (smokers vs. nonsmokers) for cancer-related genes, we found the Trk and Shp2 pathways to be differentially activated between these groups; these differences were driven by hypermethylation of the *NTRK2* and *NTRK3* genes in smokers. The differentially methylated *NTRK2* locus, located in a promoter CpG island, demonstrated an average methylation level of 36 % in the HGD/EAC samples from smokers vs. 9 % in nonsmokers. The differentially methylated *NTRK3* locus, located in the gene body, showed an average methylation of 85 % in the HGD/EAC samples of smokers compared to 62 % in nonsmokers. We previously found the aberrant methylation of *NTRK3* in 60 % of colon adenomas and 67 % of colon adenocarcinomas, suggesting *NTRK3* is a novel conditional tumor suppressor gene that is commonly inactivated in colorectal cancer by both epigenetic and genetic mechanisms [67]. *NTRK2* has also been shown to be hypermethylated in colon cancers as well as prostate cancer cell lines and cancers [68, 69]. With respect to esophageal cancer, *NTRK2* was found to have an altered allele frequency in a group of mainly esophageal squamous cell cancers, suggesting a role in esophageal cancer susceptibility and/or development [70]. The effect of DNA methylation on NTRK2 in BE and HGD/EAC is not clear at this time as its expression in BE or HGD is similar to normal esophagus based on publically available gene expression data, whereas NTRK3 is normally overexpressed in EAC (but not BE) vs. normal esophagus (expression level 1.03–1.79; www.oncomine.org).

We did not have mRNA expression data available for these samples to allow us to determine whether methylation alterations were associated with concordant changes in expression, which is a limitation of this study. In order to increase the likelihood that differences in methylation between the groups we studied were biologically relevant, we focused upon cancer-related pathways and pathways known to be involved in obesity and inflammation. Another potential limitation of this study in the EAC cases presumably contained a mix of cell types, including cancer cells, stromal cells, and inflammatory cells. We aimed to reduce the effects of cell heterogeneity by including only samples with >75 % cancer cells and focusing on genes with relatively large differences in methylation.

## Conclusions

In summary, we used a microarray-based approach to determine genome-wide methylation profiles of a collection of 81 esophageal specimens, including samples of BE, dysplastic BE, and EAC DNA. With respect to gender, BMI, and tobacco use we found numerous alterations in DNA methylation involving various regions of the genome. These results suggest that obesity and tobacco smoking influence DNA methylation in the esophagus and provide novel insights into the pathways linking these risk factors to the development of BE, dysplastic BE, and EAC.

## Methods

### Primary tissue samples and sample preparation

DNA was extracted from formalin-fixed, paraffin-embedded (FFPE) tissue cores obtained from the Department of Pathology at University Hospitals Case Medical Center using the DNAeasy blood and tissue kit (Qiagen #69504) according to the manufacturer’s instructions with minor modifications [71]. Protocols were approved by the institutional review board. All samples were reviewed by an expert gastrointestinal pathologist (JEW) prior to processing. The total number of samples prepared was: 21 Barrett’s esophagus (BE), 18 Barrett’s with low-grade dysplasia (BE + LGD), 18 Barrett’s with high-grade dysplasia (BE + HGD), and 24 with esophageal adenocarcinoma (EAC) (Additional file 5: Table S1). We also analyzed 12 cases of esophageal squamous epithelia (SQ) and compared methylation of this sample group to the EAC group to generate a list of “cancer-associated” loci.

Epithelial cell layers were identified and subsequently microdissected from glass slides. For the EAC cases, at least 75 % of each sample contained cancer in order to minimize methylation differences that might be due to cellular heterogeneity. After extraction, the DNA concentration was determined using the Quant-iT PicoGreen dsDNA assay kit (Invitrogen/Life Technologies, #P7589), and DNA quality was confirmed using the Illumina FFPE QC kit (Illumina, #WG-321-1001). Next, a total of 250 ng of each sample was sodium bisulfite converted using the EZ DNA methylation kit following the manufacturer’s protocol (ZymoResearch, #D5002), and then the DNA samples were treated with the Infinium HD FFPE DNA restore kit to repair any degraded DNA (Illumina, #WG-321-1002). Bisulfite-converted, restored DNA was submitted to the Genomics Core at the Fred Hutchinson Cancer Research Center (FHCRC) for processing, application, and scanning on the HumanMethylation450 (HM450) BeadChip following the manufacturer’s instructions (Illumina #WG-314-1003; http://www.Illumina.com).

### Genome-wide methylation arrays

HM450 BeadChips were used to analyze patterns of DNA methylation in 81 of the esophageal samples listed above. We followed our previously validated protocols for data filtering, normalization, and differential methylation analysis [61, 72] with the following modifications or clarifications: probes with detection *p* value >0.05, probes on the X chromosome, and probes containing at least one SNP with low minor allele frequency (MAF = 0) in the probe body were filtered out. After filtering, a total of 453,444 probes were available for downstream analysis. The ComBat algorithm was used to correct known batch effects across the three different microarray experiments while retaining the expected variation between the different histological tissue types [73, 74]. Data was analyzed using both “β values,” where 0.0 is equivalent to 0 % methylation and 1.0 is equivalent to 100 % and “M values” which are logarithmic scores similar to those used in gene expression microarrays. We performed clustering analysis using the 3000 most highly variable loci when considering all BE, LGD, HGD, and EAC cases assessed using the HM450 array. We used the limma and minfi Bioconductor packages to compute a refined *F*-statistic to quantify the difference in DNA methylation based on a probe’s M-value between sample types. We used a false discovery rate (FDR) *q* value to determine the significance of differentially methylated loci (DML) and considered loci to be differentially methylated if *q* < 1 × 10^−5^ [75]. Cancer-associated loci were those that showed differential methylation when comparing EAC and squamous (SQ) samples (*q* < 0.001).

The relationships of differentially methylated CpG dinucleotides to CpG islands were determined using the HM450 array annotation along with published definitions [29, 30]. Thus, a CpG island shore is the region located 0–2 kb from a CpG island, a CpG island shelf is located 2–4 kb from a CpG island, and an “open sea” is located >4 kb from a CpG island.

Differentially methylated regions (DMR) were calculated using the Bumphunter method as described by Jaffe et al. [76]. To be considered a DMR, regions had to contain at least two contiguous CpG dinucleotides that were differentially methylated with family-wise error rate (FWER) < 0.10 and *Δβ* > 0.10.

The actual vs. expected distributions of BMI and smoking-associated DML with respect to genomic location and CpG island were calculated using Pearson chi-square tests. In other words, we compared the distribution of differentially hypo- or hypermethylated loci and the distribution of all of the probes on the HM450 array with respect to genomic location/CpG island location to determine whether particular regions were enriched in hypo/hypermethylated loci.

### Gene pathway analysis

Pathway enrichment analysis of significantly differentially methylated genes between any two sample groups was performed using pathway definitions derived from the NCI Pathway Interaction Database (NCI-PID), a curated collection of known biomolecular interactions and key signaling pathways associated with cancer [77]. The enrichment analysis was performed using the hypergeometric test to evaluate if genes belonging to a given pathway were enriched among the significantly differentially methylated loci. We elected to increase the possibility that altered molecular pathways would be biologically relevant by restricting our NCI-PID analysis to only cancer-associated DML. Hochberg FDR methodology and pathways with FDR ≤ 0.05 were considered significantly methylated genes. This was followed by followed by assessment of false discovery rate using the Benjamini Hochberg correction [78]. Genes with multiple differentially methylated probes were excluded if the methylation state of any probe was inconsistent between comparison groups. Gene set enrichment analysis was performed using genes from differentially methylated groups to identify affected Gene Ontology (GO) terms and Kyoto Encyclopedia of Genes and Genomes (KEGG) pathways across different comparison groups using hypergeometric testing provided by the GOstats software [79]. Probes whose target genes were not annotated to at least one GO term in the biological process ontology were filtered out. A gene set was considered altered if its number of differentially methylated CpG sites was higher or lower than expected using a *p* value <0.05.

## Additional files

Additional file 1: Table S2. GO terms represented in DM genes comparing BE high vs. low BMI. (DOC 28 kb)
Additional file 2: Table S3. GO terms represented in DM genes comparing HGD/EAC high vs. low BMI. (DOC 28 kb)
Additional file 3: Table S4. GO terms represented in DM genes comparing BE smokers vs. nonsmokers. (DOC 28 kb)
Additional file 4: Table S5. GO terms represented in DM genes comparing HGD/EAC smokers vs. nonsmokers. (DOC 28 kb)
Additional file 5: Table S1. Samples analyzed on HM450 arrays. (DOC 122 kb)

## Abbreviations

BE: Barrett’s esophagus
BMI: Body mass index
DML: Differentially methylated loci/locus
DMR: Differentially methylated region
EAC: Esophageal adenocarcinoma
FFPE: Formalin-fixed paraffin-embedded
GO: Gene ontology
HGD: High-grade dysplasia
HM450: HumanMethylation450
KEGG: Kyoto Encyclopedia of Genes and Genomes
LGD: Low-grade dysplasia
NCI-PID: National Cancer Institute Pathway Interaction Database
SQ: Squamous esophagus
UTR: Untranslated region

## References

[1] Long E, Beales IL (2014). The role of obesity in oesophageal cancer development. Ther Adv Gastroenterol.

[2] Spechler SJ (2002). Clinical practice. Barrett’s esophagus. N Engl J Med.

[3] Werner M, Mueller J, Walch A, Hofler H (1999). The molecular pathology of Barrett’s esophagus. Histol Histopathol.

[4] Flejou JF (2005). Barrett’s oesophagus: from metaplasia to dysplasia and cancer. Gut.

[5] Reid BJ, Levine DS, Longton G, Blount PL, Rabinovitch PS (2000). Predictors of progression to cancer in Barrett’s esophagus: baseline histology and flow cytometry identify low- and high-risk patient subsets. Am J Gastroenterol.

[6] Baylin SB, Jones PA (2011). A decade of exploring the cancer epigenome—biological and translational implications. Nat Rev Cancer.

[7] Yang X, Han H, De Carvalho DD, Lay FD, Jones PA, Liang G (2014). Gene body methylation can alter gene expression and is a therapeutic target in cancer. Cancer Cell.

[8] Kaz AM, Wong CJ, Luo Y, Virgin JB, Washington MK, Willis JE, Leidner RS, Chak A, Grady WM (2011). DNA methylation profiling in Barrett’s esophagus and esophageal adenocarcinoma reveals unique methylation signatures and molecular subclasses. Epigenetics.

[9] Xu E, Gu J, Hawk ET, Wang KK, Lai M, Huang M, Ajani J, Wu X (2013). Genome-wide methylation analysis shows similar patterns in Barrett’s esophagus and esophageal adenocarcinoma. Carcinogenesis.

[10] Alvi MA, Liu X, O’Donovan M, Newton R, Wernisch L, Shannon NB, Shariff K, di Pietro M, Bergman JJ, Ragunath K (2013). DNA methylation as an adjunct to histopathology to detect prevalent, inconspicuous dysplasia and early-stage neoplasia in Barrett’s esophagus. Clin Cancer Res.

[11] Wu W, Bhagat TD, Yang X, Song JH, Cheng Y, Agarwal R, Abraham JM, Ibrahim S, Bartenstein M, Hussain Z (2013). Hypomethylation of noncoding DNA regions and overexpression of the long noncoding RNA, AFAP1-AS1, in Barrett’s esophagus and esophageal adenocarcinoma. Gastroenterology.

[12] Kaz AM, Grady WM. Epigenetic biomarkers in esophageal cancer. Cancer Lett. 2012;in press. 10.1016/j.canlet.2012.02.036 PMC3395756 22406828

[13] Eads C, Lord R, Wickramasinghe K, Long T, Kurumboor S, Bernstein L, Peters J, DeMeester S, DeMeester T, Skinner K (2001). Epigenetic patterns in the progression of esophageal adenocarcinoma. Cancer Res.

[14] Spechler SJ, Sharma P, Souza RF, Inadomi JM, Shaheen NJ (2011). American Gastroenterological Association medical position statement on the management of Barrett’s esophagus. Gastroenterology.

[15] Rubenstein JH, Thrift AP (2015). Risk factors and populations at risk: selection of patients for screening for Barrett’s oesophagus. Best Pract Res Clin Gastroenterol.

[16] Kendall BJ, Thrift AP. Unravelling the riddle of gastroesophageal reflux disease, obesity, and Barrett’s esophagus. Clin Gastroenterol Hepatol. 2015; in press. 10.1016/j.cgh.2015.08.024 26305070

[17] Hardikar S, Song X, Risques RA, Montine TJ, Duggan C, Blount PL, Reid BJ, Anderson GL, Kratz M, White E (2015). Obesity and inflammation markers in relation to leukocyte telomere length in a cross-sectional study of persons with Barrett’s esophagus. BMC Obesity.

[18] Alegria-Torres JA, Baccarelli A, Bollati V (2011). Epigenetics and lifestyle. Epigenomics.

[19] Drummond EM, Gibney ER (2013). Epigenetic regulation in obesity. Curr Opin Clin Nutr Metab Care.

[20] van Dijk SJ, Molloy PL, Varinli H, Morrison JL, Muhlhausler BS, Members of Epi, S (2015). Epigenetics and human obesity. Int J Obes.

[21] Ding X, Zheng D, Fan C, Liu Z, Dong H, Lu Y, Qi K (2015). Genome-wide screen of DNA methylation identifies novel markers in childhood obesity. Gene.

[22] Hair BY, Troester MA, Edmiston SN, Parrish EA, Robinson WR, Wu MC, Olshan AF, Swift-Scanlan T, Conway K (2015). Body mass index is associated with gene methylation in estrogen receptor-positive breast tumors. Cancer Epidemiol Biomarkers Prev.

[23] Enokida H, Shiina H, Urakami S, Terashima M, Ogishima T, Li LC, Kawahara M, Nakagawa M, Kane CJ, Carroll PR (2006). Smoking influences aberrant CpG hypermethylation of multiple genes in human prostate carcinoma. Cancer.

[24] Belinsky SA, Palmisano WA, Gilliland FD, Crooks LA, Divine KK, Winters SA, Grimes MJ, Harms HJ, Tellez CS, Smith TM (2002). Aberrant promoter methylation in bronchial epithelium and sputum from current and former smokers. Cancer Res.

[25] Wan ES, Qiu W, Baccarelli A, Carey VJ, Bacherman H, Rennard SI, Agusti A, Anderson W, Lomas DA, Demeo DL (2012). Cigarette smoking behaviors and time since quitting are associated with differential DNA methylation across the human genome. Hum Mol Genet.

[26] Zeilinger S, Kuhnel B, Klopp N, Baurecht H, Kleinschmidt A, Gieger C, Weidinger S, Lattka E, Adamski J, Peters A (2013). Tobacco smoking leads to extensive genome-wide changes in DNA methylation. PLoS One.

[27] Chandar AK, Iyer PG (2015). Role of obesity in the pathogenesis and progression of Barrett’s esophagus. Gastroenterol Clin N Am.

[28] Rubenstein JH, Shaheen NJ (2015). Epidemiology, diagnosis, and management of esophageal adenocarcinoma. Gastroenterology.

[29] Irizarry RA, Ladd-Acosta C, Wen B, Wu Z, Montano C, Onyango P, Cui H, Gabo K, Rongione M, Webster M (2009). The human colon cancer methylome shows similar hypo- and hypermethylation at conserved tissue-specific CpG island shores. Nat Genet.

[30] Bibikova M, Barnes B, Tsan C, Ho V, Klotzle B, Le JM, Delano D, Zhang L, Schroth GP, Gunderson KL (2011). High density DNA methylation array with single CpG site resolution. Genomics.

[31] Yuan R, Wang K, Hu J, Yan C, Li M, Yu X, Liu X, Lei J, Guo W, Wu L (2014). Ubiquitin-like protein FAT10 promotes the invasion and metastasis of hepatocellular carcinoma by modifying beta-catenin degradation. Cancer Res.

[32] Howe LR, Subbaramaiah K, Hudis CA, Dannenberg AJ (2013). Molecular pathways: adipose inflammation as a mediator of obesity-associated cancer. Clin Cancer Res.

[33] Zeng L, Perks CM, Holly JM (2015). IGFBP-2/PTEN: a critical interaction for tumours and for general physiology?. Growth Horm IGF Res.

[34] Mauro L, Naimo GD, Ricchio E, Panno ML, Ando S (2015). Cross-talk between adiponectin and IGF-IR in breast cancer. Front Oncol.

[35] Jiang N, Sun R, Sun Q (2014). Leptin signaling molecular actions and drug target in hepatocellular carcinoma. Drug Des Devel Ther.

[36] Fan H, Zhao H, Pang L, Liu L, Zhang G, Yu F, Liu T, Xu C, Xiao Y, Li X (2015). Systematically prioritizing functional differentially methylated regions (fDMRs) by integrating multi-omics data in colorectal cancer. Sci Rep.

[37] Kretzmer H, Bernhart SH, Wang W, Haake A, Weniger MA, Bergmann AK, Betts MJ, Carrillo-de-Santa-Pau E, Doose G, Gutwein J. et al. DNA methylome analysis in Burkitt and follicular lymphomas identifies differentially methylated regions linked to somatic mutation and transcriptional control. Nat Genet. 2015; in press. 10.1038/ng.3413 PMC5444523 26437030

[38] El-Maarri O, Becker T, Junen J, Manzoor SS, Diaz-Lacava A, Schwaab R, Wienker T, Oldenburg J (2007). Gender specific differences in levels of DNA methylation at selected loci from human total blood: a tendency toward higher methylation levels in males. Hum Genet.

[39] Fuke C, Shimabukuro M, Petronis A, Sugimoto J, Oda T, Miura K, Miyazaki T, Ogura C, Okazaki Y, Jinno Y (2004). Age related changes in 5-methylcytosine content in human peripheral leukocytes and placentas: an HPLC-based study. Ann Hum Genet.

[40] Sarter B, Long TI, Tsong WH, Koh WP, Yu MC, Laird PW (2005). Sex differential in methylation patterns of selected genes in Singapore Chinese. Hum Genet.

[41] Breitling LP, Yang R, Korn B, Burwinkel B, Brenner H (2011). Tobacco-smoking-related differential DNA methylation: 27 K discovery and replication. Am J Hum Genet.

[42] Levy P, Ripoche H, Laurendeau I, Lazar V, Ortonne N, Parfait B, Leroy K, Wechsler J, Salmon I, Wolkenstein P (2007). Microarray-based identification of tenascin C and tenascin XB, genes possibly involved in tumorigenesis associated with neurofibromatosis type 1. Clin Cancer Res.

[43] Hsu MK, Wu IC, Cheng CC, Su JL, Hsieh CH, Lin YS, Chen FC. Triple-layer dissection of the lung adenocarcinoma transcriptome—regulation at the gene, transcript, and exon levels. Oncotarget. 2015; in press. 10.18632/oncotarget.4810 PMC4745690 26356813

[44] Klausen C, Leung PC, Auersperg N (2009). Cell motility and spreading are suppressed by HOXA4 in ovarian cancer cells: possible involvement of beta1 integrin. Mol Cancer Res.

[45] Musialik E, Bujko M, Kober P, Grygorowicz MA, Libura M, Przestrzelska M, Juszczynski P, Borg K, Florek I, Jakobczyk M (2015). Promoter DNA methylation and expression levels of HOXA4, HOXA5 and MEIS1 in acute myeloid leukemia. Mol Med Rep.

[46] Phelan JD, Saba I, Zeng H, Kosan C, Messer MS, Olsson HA, Fraszczak J, Hildeman DA, Aronow BJ, Moroy T (2013). Growth factor independent-1 maintains Notch1-dependent transcriptional programming of lymphoid precursors. PLoS Genet.

[47] Khandanpour C, Moroy T (2013). Growth factor independence 1 (Gfi1) as a regulator of p53 activity and a new therapeutical target for ALL. Oncotarget.

[48] Agarwal R, Mori Y, Cheng Y, Jin Z, Olaru AV, Hamilton JP, David S, Selaru FM, Yang J, Abraham JM (2009). Silencing of claudin-11 is associated with increased invasiveness of gastric cancer cells. PLoS One.

[49] Agarwal A, Polineni R, Hussein Z, Vigoda I, Bhagat TD, Bhattacharyya S, Maitra A, Verma A (2012). Role of epigenetic alterations in the pathogenesis of Barrett’s esophagus and esophageal adenocarcinoma. Int J Clin Exp Pathol.

[50] Tang JT, Wang ZH, Fang JY (2015). Assessing the potential value of long interspersed element-1 hypomethylation in colorectal cancer: evidence from retrospective studies. OncoTargets Ther.

[51] Ziller MJ, Gu H, Muller F, Donaghey J, Tsai LT, Kohlbacher O, De Jager PL, Rosen ED, Bennett DA, Bernstein BE (2013). Charting a dynamic DNA methylation landscape of the human genome. Nature.

[52] Baylin SB, Herman JG, Graff JR, Vertino PM, Issa JP (1998). Alterations in DNA methylation: a fundamental aspect of neoplasia. Adv Cancer Res.

[53] Martinowich K, Hattori D, Wu H, Fouse S, He F, Hu Y, Fan G, Sun YE (2003). DNA methylation-related chromatin remodeling in activity-dependent BDNF gene regulation. Science.

[54] Tsai MM, Wang CS, Tsai CY, Chen CY, Chi HC, Tseng YH, Chung PJ, Lin YH, Chung IH, Chen CY (2014). MicroRNA-196a/-196b promote cell metastasis via negative regulation of radixin in human gastric cancer. Cancer Lett.

[55] Reid BJ (2010). Early events during neoplastic progression in Barrett’s esophagus. Cancer Biomark.

[56] Reid BJ (2001). p53 and neoplastic progression in Barrett’s esophagus. Am J Gastroenterol.

[57] Dolan K, Morris AI, Gosney JR, Field JK, Sutton R (2003). Loss of heterozygosity on chromosome 17p predicts neoplastic progression in Barrett’s esophagus. J Gastroenterol Hepatol.

[58] Feinberg AP, Irizarry RA, Fradin D, Aryee MJ, Murakami P, Aspelund T, Eiriksdottir G, Harris TB, Launer L, Gudnason V (2010). Personalized epigenomic signatures that are stable over time and covary with body mass index. Sci Transl Med.

[59] Demerath EW, Guan W, Grove ML, Aslibekyan S, Mendelson M, Zhou YH, Hedman AK, Sandling JK, Li LA, Irvin MR (2015). Epigenome-wide association study (EWAS) of BMI, BMI change and waist circumference in African American adults identifies multiple replicated loci. Hum Mol Genet.

[60] Dick KJ, Nelson CP, Tsaprouni L, Sandling JK, Aissi D, Wahl S, Meduri E, Morange PE, Gagnon F, Grallert H (2014). DNA methylation and body-mass index: a genome-wide analysis. Lancet.

[61] Kaz AM, Wong CJ, Dzieciatkowski S, Luo Y, Schoen RE, Grady WM. Patterns of DNA methylation in the normal colon vary by anatomical location, gender, and age. Epigenetics. 2014;9:492–502. 10.4161/epi.27650 PMC4121360 24413027

[62] Inoshita M, Numata S, Tajima A, Kinoshita M, Umehara H, Yamamori H, Hashimoto R, Imoto I, Ohmori T (2015). Sex differences of leukocytes DNA methylation adjusted for estimated cellular proportions. Biol Sex Differ.

[63] Liu J, Morgan M, Hutchison K, Calhoun VD (2010). A study of the influence of sex on genome wide methylation. PLoS One.

[64] Runge TM, Abrams JA, Shaheen NJ (2015). Epidemiology of Barrett’s esophagus and esophageal adenocarcinoma. Gastroenterol Clin N Am.

[65] Toh Y, Oki E, Ohgaki K, Sakamoto Y, Ito S, Egashira A, Saeki H, Kakeji Y, Morita M, Sakaguchi Y (2010). Alcohol drinking, cigarette smoking, and the development of squamous cell carcinoma of the esophagus: molecular mechanisms of carcinogenesis. Int J Clin Oncol.

[66] Xu XC (2009). Risk factors and gene expression in esophageal cancer. Methods Mol Biol.

[67] Luo Y, Kaz AM, Kanngurn S, Welsch P, Morris SM, Wang J, Lutterbaugh JD, Markowitz SD, Grady WM (2013). NTRK3 is a potential tumor suppressor gene commonly inactivated by epigenetic mechanisms in colorectal cancer. PLoS Genet.

[68] Ahmed D, Danielsen SA, Aagesen TH, Bretthauer M, Thiis-Evensen E, Hoff G, Rognum TO, Nesbakken A, Lothe RA, Lind GE (2012). A tissue-based comparative effectiveness analysis of biomarkers for early detection of colorectal tumors. Clin Transl Gastroenterol.

[69] Yamada Y, Toyota M, Hirokawa Y, Suzuki H, Takagi A, Matsuzaki T, Sugimura Y, Yatani R, Shiraishi T, Watanabe M (2004). Identification of differentially methylated CpG islands in prostate cancer. Int J Cancer.

[70] Chen J, Guo L, Peiffer DA, Zhou L, Chan OT, Bibikova M, Wickham-Garcia E, Lu SH, Zhan Q, Wang-Rodriguez J (2008). Genomic profiling of 766 cancer-related genes in archived esophageal normal and carcinoma tissues. Int J Cancer.

[71] Moinova H, Leidner RS, Ravi L, Lutterbaugh J, Barnholtz-Sloan JS, Chen Y, Chak A, Markowitz SD, Willis JE (2012). Aberrant vimentin methylation is characteristic of upper gastrointestinal pathologies. Cancer Epidemiol Biomarkers Prev.

[72] Luo Y, Wong CJ, Kaz AM, Dzieciatkowski S, Carter KT, Morris SM, Wang J, Willis JE, Makar KW, Ulrich CM (2014). Differences in DNA methylation signatures reveal multiple pathways of progression from adenoma to colorectal cancer. Gastroenterology.

[73] Johnson WE, Li C, Rabinovic A (2007). Adjusting batch effects in microarray expression data using empirical Bayes methods. Biostatistics.

[74] Leek JT, Scharpf RB, Bravo HC, Simcha D, Langmead B, Johnson WE, Geman D, Baggerly K, Irizarry RA (2010). Tackling the widespread and critical impact of batch effects in high-throughput data. Nat Rev Genet.

[75] Storey JD (2003). The positive false discovery rate: a Bayesian interpretation and the q-value. Ann Stat.

[76] Jaffe AE, Murakami P, Lee H, Leek JT, Fallin MD, Feinberg AP, Irizarry RA (2012). Bump hunting to identify differentially methylated regions in epigenetic epidemiology studies. Int J Epidemiol.

[77] Schaefer CF, Anthony K, Krupa S, Buchoff J, Day M, Hannay T, Buetow KH (2009). PID: the Pathway Interaction Database. Nucleic Acids Res.

[78] Benjamini Y, Drai D, Elmer G, Kafkafi N, Golani I (2001). Controlling the false discovery rate in behavior genetics research. Behav Brain Res.

[79] Falcon S, Gentleman R (2007). Using GOstats to test gene lists for GO term association. Bioinformatics.

