# Erratum to: Global DNA methylation patterns in Barrett’s esophagus, dysplastic Barrett’s, and esophageal adenocarcinoma are associated with BMI, gender, and tobacco use

**DOI:** 10.1186/s13148-017-0324-8

**Published:** 2017-03-01

**Authors:** Andrew M. Kaz, Chao-Jen Wong, Vinay Varadan, Joseph E. Willis, Amitabh Chak, William M. Grady

**Affiliations:** 1 Gastroenterology Section, VA Puget Sound Health Care System, Seattle, WA 98108 USA; 2 Clinical Research Division, Fred Hutchinson Cancer Research Center, Seattle, WA 98109 USA; 3 Department of Medicine, University of Washington School of Medicine, Seattle, WA 98195 USA; 4 Case Comprehensive Cancer Center, Case Western Reserve University, Cleveland, OH 44106 USA; 5 Department of Pathology, Case Western Reserve University School of Medicine, Cleveland, OH 44106 USA; 6 Division of Gastroenterology, Case Western Reserve University School of Medicine, Cleveland, OH 44106 USA

## Erratum

Following publication of this article [1], it has come to our attention that the following publication should have acknowledged the DeGregorio Family Foundation and the Price Family Foundation as providing funding for the studies and for supporting the work.
