# Supplementary material for: Global DNA methylation patterns in Barrett’s esophagus, dysplastic Barrett’s, and esophageal adenocarcinoma are associated with BMI, gender, and tobacco use
Source: Clin Epigenetics. 2016 Oct 27;8:111. doi: 10.1186/s13148-016-0273-7 (PMC5082363; doi:10.1186/s13148-016-0273-7)
Supplement: Additional file 4: Table S5. — GO terms represented in DM genes comparing HGD/EAC smokers vs. nonsmokers. (DOC 28 kb) [file 13148_2016_273_MOESM4_ESM.doc]

Additional file 4: Table S5: GO terms represented in DM genes comparing HGD/EAC smokers vs

nonsmokers

| **Tissue** | **Total No. DML** | **Promoter DML (%)** | **Intragenic DML (%)** | **Intergenic DML (%)** | **CpG island DML (%)** | **CpG shore DML (%)** | **Cancer-associated DML (%)** |
| --- | --- | --- | --- | --- | --- | --- | --- |
| BE | 86 | 25 (29%) | 43 (50%) | 18 (21%) | 23 (27%) | 28  (32%) | 21  (24%) |
| HGD/  EAC | 802 | 152 (19%) | 417 (52%) | 233 (29%) | 279  (35%) | 395  (49%) | 79  (10%) |
