# Supplementary material for: Global DNA methylation patterns in Barrett’s esophagus, dysplastic Barrett’s, and esophageal adenocarcinoma are associated with BMI, gender, and tobacco use
Source: Clin Epigenetics. 2016 Oct 27;8:111. doi: 10.1186/s13148-016-0273-7 (PMC5082363; doi:10.1186/s13148-016-0273-7)
Supplement: Additional file 5: Table S1. — Samples analyzed on HM450 arrays. (DOC 122 kb) [file 13148_2016_273_MOESM5_ESM.doc]

Additional file 5: Table S1: Samples analyzed on HM450 arrays

| **Patient ID** | **Histology** | **Age/Gender** | **Race** | **BMI** | **Smoker** |
| --- | --- | --- | --- | --- | --- |
| 1 | EAC | 64 M | W | 39.2 | - |
| 1 | SQ | 64 M | W | 39.2 | - |
| 3 | HGD | 52 M | W | - | Y |
| 3 | EAC | 52 M | W | - | Y |
| 3 | LGD | 52 M | W | - | Y |
| 3 | SQ | 52 M | W | - | Y |
| 4 | EAC | 76 F | W | - | Y |
| 4 | SQ | 76 F | W |  | Y |
| 5 | EAC | 49 F | W | 28.8 | N |
| 7 | LGD | 67 M | W | - | - |
| 7 | SQ | 67 M | W | - | - |
| 8 | EAC | 56 M | - | - | - |
| 8 | HGD | 56 M | - | - | - |
| 9 | EAC | 65 M | W | - | - |
| 10 | HGD | 82 M | W | - | - |
| 10 | BE | 82 M | W |  | - |
| 10 | HGD | 82 M | W |  | - |
| 11 | EAC | 82 M | - |  | - |
| 12 | EAC | 77 M | W | - | Y |
| 13 | EAC | 79 M | W | - | - |
| 13 | BE | 79 M | W |  | - |
| 14 | EAC | 64 M | W | - | N |
| 14 | BE | 64 M | W |  | N |
| 15 | EAC | 48 M | W | 38.7 | Y |
| 16 | HGD | 56 F | W | 24.6 | Y |
| 18 | BE | 78 M | W | - | - |
| 21 | LGD | 79 M | AA | 30.0 | - |
| 21 | EAC | 79 M | AA | 30.0 | - |
| 21 | HGD | 79 M | AA | 30.0 | - |
| 22 | HGD | 71 M | W | 30.0 | N |
| 22 | EAC | 71 M | W | 30.0 | N |
| 22 | BE | 71 M | W | 30.0 | N |
| 22 | SQ | 71 M | W | 30.0 | N |
| 23 | EAC | 88 M | W | 23.6 | Y |
| 23 | SQ | 88 M | W | 23.6 | Y |
| 24 | BE | 83 M | W | - | - |
| 24 | HGD/EAC | 83 M | W | - | - |
| 24 | LGD | 83 M | W | - | - |
| 24 | EAC | 83 M | W | - | - |
| 26 | HGD/EAC | 53 F | W | - | N |
| 26 | BE | 53 F | W | - | N |
| 27 | SQ | 69 F | W | - | Y |
| 28 | HGD | 72 M | W | - | Y |
| 28 | EAC | 72 M | W | - | Y |
| 28 | LGD | 72 M | W | - | Y |
| 28 | SQ | 72 M | W | - | Y |
| 29 | BE | 46 M | W | 26.0 | N |
| 31 | EAC | 74 M | W | - | - |
| 32 | EAC | 54 M | W | - | - |
| 34 | EAC | 68 M | W | - | Y |
| 34 | SQ | 68 M | W | - | Y |
| 35 | SQ | 55 M | W | 25.1 | - |
| 37 | LGD | 77 M | W | 25.0 | Y |
| 37 | EAC | 77 M | W | 25.0 | Y |
| 37 | HGD | 77 M | W | 25.0 | Y |
| 37 | BE | 77 M | W | 25.0 | Y |
| 37 | SQ | 77 M | W | 25.0 | Y |
| 38 | EAC | 70 M | W | - | - |
| 39 | HGD | 74 M | W | - | N |
| 40 | HGD | 62 M | W | 39.9 | Y |
| 41 | BE | 63 M | W | - | N |
| 41 | EAC | 63 M | W | - | N |
| 42 | HGD | 76 F | W | - | Y |
| 42 | EAC | 76 F | W | - | Y |
| 44 | BE | 73 M | W | 28.7 | Y |
| 45 | HGD | 73 M | W | 20.6 | - |
| 46 | HGD | 93 M | W | 21.9 | Y |
| 50 | BE | 43 F | W | 31.0 | Y |
| 50 | BE | 43 F | W | 31.0 | Y |
| 50 | SQ | 43 F | W | 31.0 | Y |
| 53 | BE | 52 M | W | 37.2 | Y |
| 54 | BE | 69 M | W | 27.4 | Y |
| 55 | BE | 68 M | W | 28.9 | Y |
| 58 | BE | 67 F | - | - | - |
| 59 | BE | 71 F | W | - | Y |
| 60 | BE | 40 M | W | 27.7 | N |
| 64 | BE | 76 M | W | - | Y |
| 65 | BE | 70 M | W | 24.4 | N |
| 67 | LGD | 78 M | W | 26.1 | Y |
| 69 | LGD | 55 M | W | 33.1 | Y |
| 70 | LGD | 69 M | W | 41.2 | - |
| 72 | LGD | 66 M | W | 42.8 | Y |
| 72 | EAC | 66 M | W | 42.8 | Y |
| 73 | LGD | 51 M | W | 30.7 | Y |
| 74 | LGD | 85 M | W | 34.0 | Y |
| 76 | LGD | 48 M | W | 30.4 | Y |
| 77 | LGD | 73 M | W | 28.2 | - |
| 80 | HGD | 71 M | W | 23.7 | Y |
| 81 | LGD | 70 M | W | 30.0 | Y |
| 84 | LGD | 71 M | W | 29.5 | - |
| 86 | LGD | 71 M | W | 22.7 | Y |
| 88 | LGD | 93 M | W | 21.9 | Y |
| 89 | HGD | 69 M | W | 34.1 | Y |

Each patient ID refers to a unique individual; note in some cases multiple sample types

were obtained from the same individual. BMI=body mass index. “-“ denotes data not

available.
